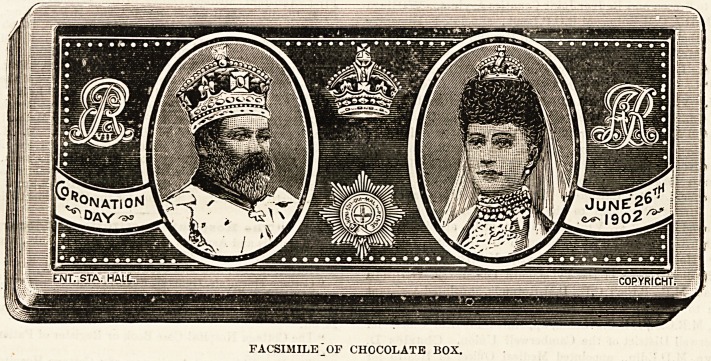# The King's Chocolate

**Published:** 1902-06-21

**Authors:** 


### THE KING'S CHOCOLATE.

A specimen of the chocolate which will be given to the guests at the King's Coronation Dinner, has been submitted to us. It is made by Messrs. Rowntree, one of the firms who supplied the "Queen's Chocolate" to the troops in South Africa two and a half years ago, and it has a pleasant and delicate flavour. The chocolate is packed in small tin cases, on the cover of which are coloured portraits of the King and Queen; the boxes are manufactured by Messrs. Barringer, Wallis, and Manners, Limited, of Mansfield, who made a large number of the Queen's Chocolate boxes. A finished tin was submitted to his Majesty and approved by him.

FACSIMILE OF CHOCOLATE BOX.

The number of guests for the King's dinners is 500,000, and 60,000 stewards will wait upon them. Messrs. Rowntree are supplying a sufficient number of these boxes of chocolate to allow of one being given to each guest and the steward; these they have presented to the King, who has graciously accepted them for presentation in his name to the poor of London, who will partake of his hospitality. It is possible that some of the chocolate, like some that was sent to South Africa, may not be eaten at all, but saved up as an heirloom, or shown to less fortunate country cousins in the future; and, at any rate, the box in which it is packed will be preserved as a souvenir of the event.

## Figures and Tables

**Figure f1:**